# Coordinate Regulation of Mature Dopaminergic Axon Morphology by Macroautophagy and the PTEN Signaling Pathway

**DOI:** 10.1371/journal.pgen.1003845

**Published:** 2013-10-03

**Authors:** Keiichi Inoue, Joanne Rispoli, Lichuan Yang, David MacLeod, M. Flint Beal, Eric Klann, Asa Abeliovich

**Affiliations:** 1 Departments of Pathology and Neurology, Taub Institute, Columbia University Medical Center, New York, New York, United States of America; 2 Department of Neurology and Neuroscience, Weill Cornell Medical College of Cornell University, New York, New York, United States of America; 3 Center for Neural Science, New York University, New York, New York, United States of America; Stanford University School of Medicine, United States of America

## Abstract

Macroautophagy is a conserved mechanism for the bulk degradation of proteins and organelles. Pathological studies have implicated defective macroautophagy in neurodegeneration, but physiological functions of macroautophagy in adult neurons remain unclear. Here we show that Atg7, an essential macroautophagy component, regulates dopaminergic axon terminal morphology. Mature Atg7-deficient midbrain dopamine (DA) neurons harbored selectively enlarged axonal terminals. This contrasted with the phenotype of DA neurons deficient in Pten – a key negative regulator of the mTOR kinase signaling pathway and neuron size – that displayed enlarged soma but unaltered axon terminals. Surprisingly, concomitant deficiency of both Atg7 and Pten led to a dramatic enhancement of axon terminal enlargement relative to Atg7 deletion alone. Similar genetic interactions between Atg7 and Pten were observed in the context of DA turnover and DA-dependent locomotor behaviors. These data suggest a model for morphological regulation of mature dopaminergic axon terminals whereby the impact of mTOR pathway is suppressed by macroautophagy.

## Author Summary

Macroautophagy is a major recycling pathway in cells, and its dysfunction is associated with neurological disorders including Alzheimer's disease, Parkinson's disease, and frontotemporal dementia. Here we show that Atg7, an essential component of macroautophagy, regulates mature dopaminergic axon terminal morphology in coordination with the well-described role of the PI3K pathway. Deficiency of Pten, a negative regulator of the PI3K/mTOR pathway, leads primarily to enlarged dopaminergic cell soma but normal-appearing axonal terminals, whereas Atg7 deficiency primarily induces enlarged axonal terminals. Atg7 and Pten double deficiency leads to further axon terminal enlargement, suggesting that Atg7 deficiency unmasks the impact of PI3K/mTOR pathway on mature dopaminergic axon terminals. In addition, we show that Atg7 and Pten coordinately regulate striatal dopamine turnover and dopamine-dependent motor behaviors. Taken together, these data support a novel role for Atg7-dependent macroautophagy in the regulation of dopaminergic axon terminal morphology, in coordination with the PI3K/mTOR pathway.

## Introduction

Macroautophagy is an intracellular protein degradation mechanism that engulfs cytoplasmic constituents and entire organelles within double-membrane vesicles and delivers these to lysosomes [1], [2]. Genetic deletion of the essential macroautophagy components *Atg5* or *Atg7*, during mouse central nervous system (CNS) development, leads to neuronal loss and inclusion formation [3], [4]. Furthermore, Atg7 deficiency confined to cerebellar Purkinje cells leads to dystrophic axons and subsequent cell death within several weeks [5], [6]. In addition to pathological roles, protein degradation pathways may also play important physiological functions in neurons. Several studies have underscored the role of cytoplasmic protein degradation through the ubiquitin-proteosome system (UPS) in the regulation of neuronal morphology and function [7]. However, the role of macroautophagy in this context is unclear.

A key regulator of neuronal morphology and size is PTEN (phosphatase and tensin homolog) [8], [9], an intracellular lipid phosphatase that opposes phosphatidylinositide 3-kinase (PI3K) activity. Deletion of Pten in mouse hippocampus neurons disinhibits the mTOR (mammalian target of rapamycin) signaling pathway, leading to morphological enlargement as well as altered synaptic plasticity and plasticity-related behaviors [10]–[13]. mTOR is a major activator of protein translation as well as a key inhibitor of macroautophagy, but the relative contribution of these different downstream mechanisms on neuronal size remain unclear. Thus, we hypothesized that altered macroautophagy may play a role in the regulation of neuronal morphology and function in the mammalian CNS, either downstream of or in conjunction with the PTEN/PI3K/mTOR pathway.

Cell size regulation in the mammalian CNS appears to be highly dependent on developmental stage, cell type, and subcellular region. The deletion of Pten at embryonic stages leads to a profound enlargement of neurons and glia [10], [11], whereas the deletion at later developmental stages – such as postnatally or in young adult animals – appears to have a lesser impact [12], [13]. Postnatal deletion of Pten in certain neuronal subtypes fails to alter the size of neurite processes [13]–[15], suggesting selective subcellular and developmental regulatory mechanisms. For instance, deletion of Pten in post-mitotic midbrain dopamine (DA) neurons leads to soma hypertrophy, but axonal terminal morphology appears unaltered [14], [15].

Here we investigated the role of Atg7 and macroautophagy in the regulation of mature midbrain DA neuron morphology, and contrasted this with the impact of the PI3K/mTOR pathway. Atg7 deficiency in mature DA neurons led to enlargement of axon terminals, whereas DA neuron soma size was only modestly altered. This phenotype was distinct from that of Pten-deficient mature midbrain DA neurons, which showed robust soma hypertrophy but no significant alteration at axon terminals. Mature midbrain DA neurons deficient in both Pten and Atg7 showed a dramatic enhancement of the axon terminal enlargement phenotype seen with Atg7 deficiency alone. A similar synergistic genetic interaction was similarly observed between *Pten* and *Atg7* in the context of DA metabolism (turnover) in the striatum, and with respect to DA-associated locomotor behaviors. Taken together, these data support a model whereby macroautophagy activity normally limits the impact of the PTEN/PI3K/mTOR pathway, such that mature dopaminergic axon terminals are unaffected by Pten loss. However in the absence of macroautophagy activity, the impact of the PI3K/mTOR pathway is unmasked and leads to a profound further enlargement.

## Results

### Generation of Atg7 deficient mice specifically within mature midbrain DA neurons

**Figure 1:**
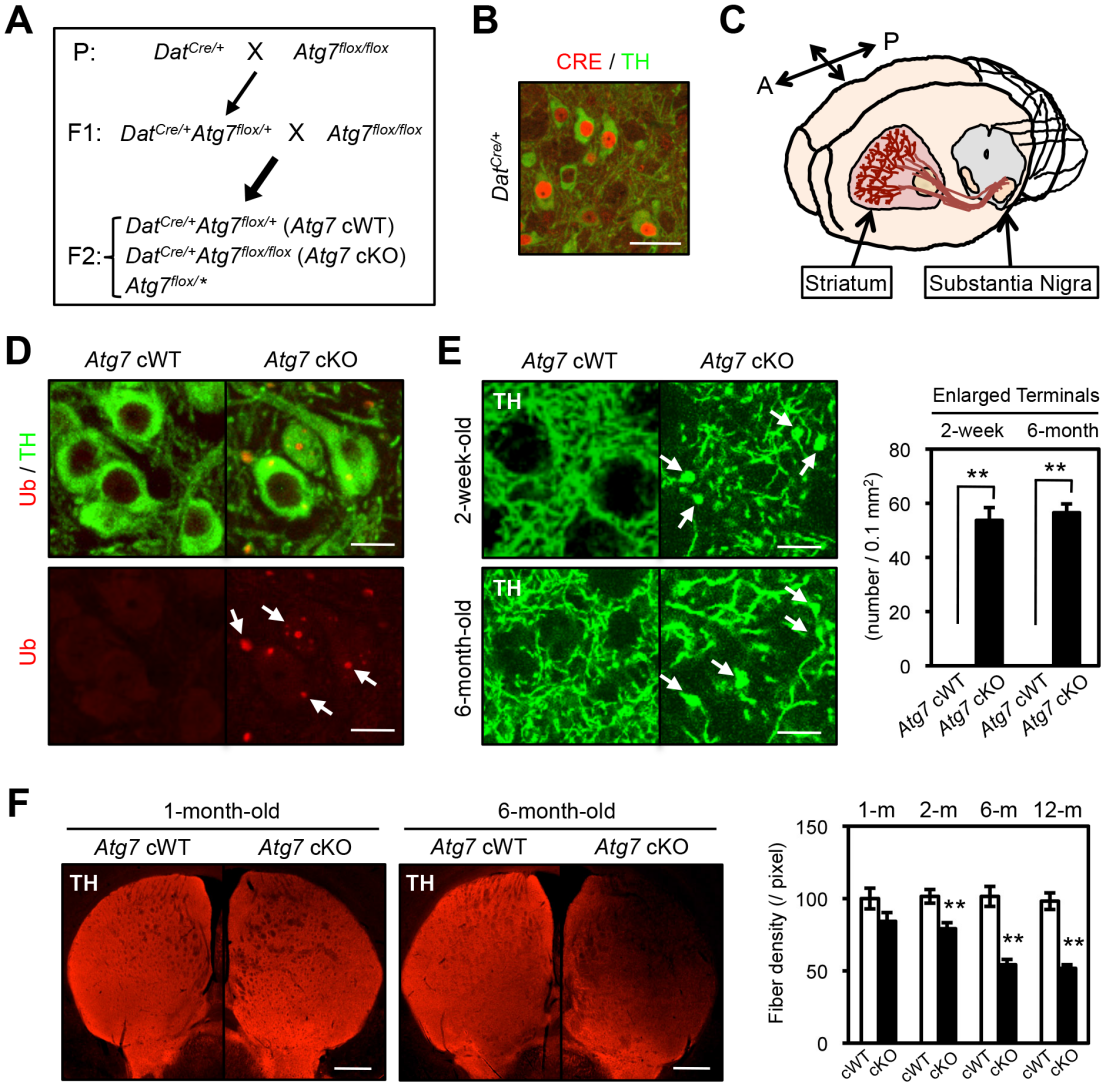
Enlarged axon terminals in TH-positive DA neurons of *Atg7* cKO mice. (**A**) Schema of mouse mating to obtain Atg7-deficient mice. *Atg7* cKO mice and the littermate *Dat^Cre/+^* (*Atg7* cWT) animals were used for the analyses. Animals lacking *Cre* (Atg7^flox/^*) were not used in this study. Asterisk indicates ‘+’ or ‘flox’. (**B**) CRE immunohistochemistry in 4-week-old *Dat^Cre/+^* midbrain. Nuclear CRE-positive staining (red) was seen in TH-positive (green) DA neurons, but not other cell types in the substantia nigra, not elsewhere in the CNS of cKO mice, and not in control *Dat^+/+^* brain [59]. Bar, 20 µm. (**C**) TH-positive DA neurons in the midbrain substantia nigra project their axons into the striatum region. A, anterior; P, posterior. (**D**) Grossly normal appearance of *Atg7* cKO midbrain DA neurons at the age of 1-month-old. Numerous Ub- and p62-positive [16] inclusions in *Atg7* cKO soma and dendrites. Bar, 10 µm. (**E**) Enlarged TH-positive axon terminals (green) in the striatum of *Atg7* cKO mice (arrows) at ages of 2-weeks and 6-months. Bars, 20 µm. (right) Quantification of the density of enlarged axon terminals. TH-positive axonal terminal enlargement in *Atg7* cKO mouse striatum was apparent from 2-week of age, and did not progress with age (6-month). White, *Atg7* cWT; Black, *Atg7* cKO. n = 6 per group. **, *p*<0.01. (**F**) The fiber density of dopaminergic axon terminals in the striatum of *Atg7* cKO mice at different ages. Dopaminergic axon terminals were visualized by anti-TH antibody staining (red). Bars, 500 µm. (right) Quantification of the density of TH-positive axonal shafts. The values were normalized by the fiber density of 1-month-old cWT animals. White, *Atg7* cWT; Black, *Atg7* cKO. n = 5∼6 per group. **, *p*<0.01.

Mice deficient in *Atg7* specifically within mature midbrain DA neurons (*Dat^Cre/+^Atg7^flox/flox^* [*Atg7* cKO]) were generated [16] by interbreeding mice that express Cre recombinase (CRE) under the dopamine transporter promoter (*Dat^Cre/+^*, Figure 1B) [17] with mice that harbor *Atg7* allele flanked by loxP sites (*Atg7^flox/flox^*, Figure 1A) [18]. Mutant animals appeared grossly normal and survival was not significantly altered (data not shown). To confirm alteration in macroautophagy activity, we initially quantified the lipidated conversion of LC3 (LC3-II), a marker for autophagosome formation that is dependent on Atg7, in crude extracts from 2-month-old midbrain tissues including substantia nigra (Figure 1C). LC3 conversion was significantly reduced in *Atg7* cKO mouse midbrains (Figure S1A). Reduction in LC3-II was only partial, likely due to ATG7 activity in non-dopaminergic midbrain cells. *Atg7* cKO mice displayed a normal number [16] and gross appearance of tyrosine hydroxylase (TH)-positive DA neurons in the substantia nigra at 1-month of age (Figure 1D). As shown in our prior paper, the number of TH-positive DA neurons, however, declined progressively from 2 month of age, and approximately 50% of TH-positive cells were lost by 1 year of age [16]. Furthermore, ubiquitin (Ub)- and p62-positive inclusions were apparent in *Atg7* cKO DA neuron cell bodies and dendrites from 1-month of age (Figure 1D) [16].

### Midbrain DA neurons lacking Atg7 display enlarged axon terminals

**Figure 2:**
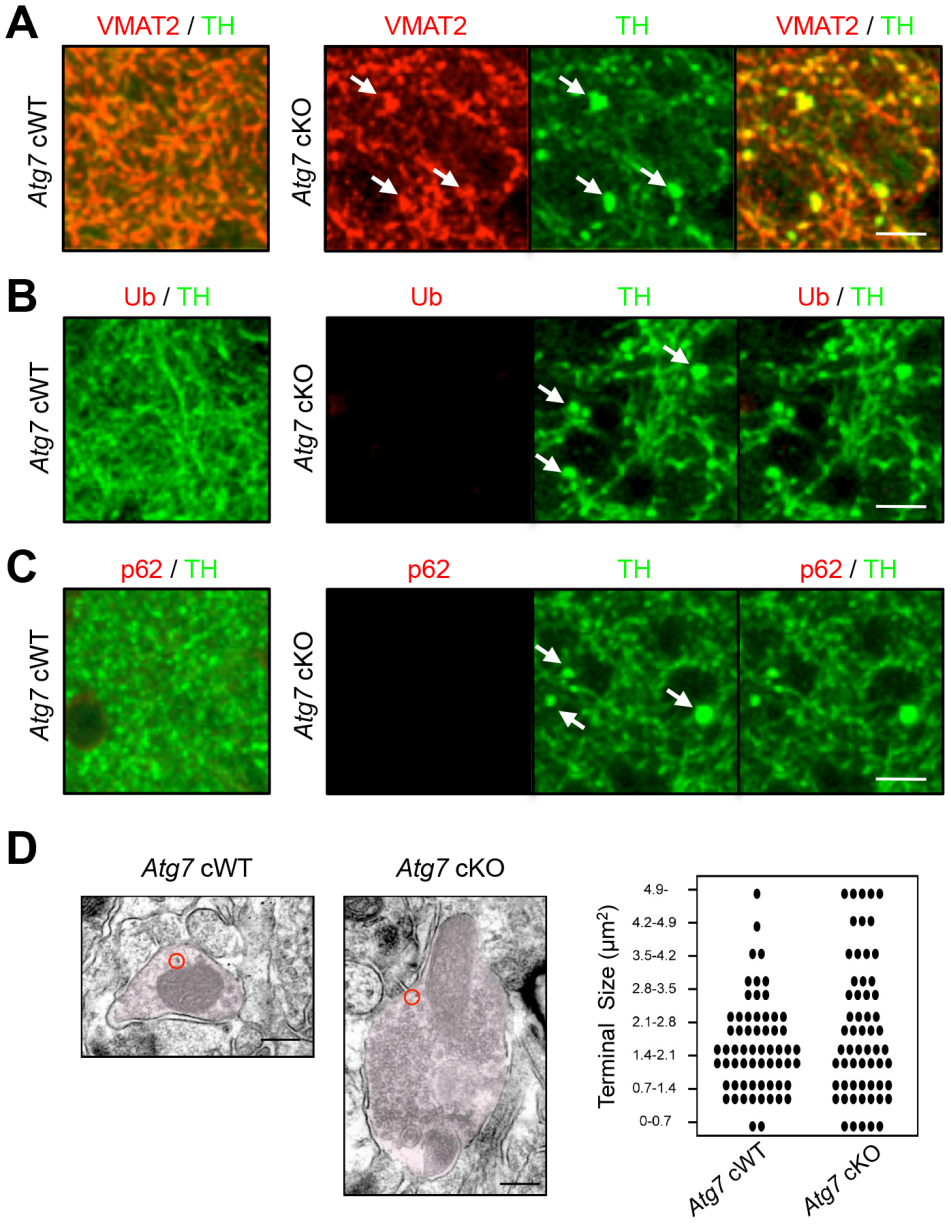
Characterization of enlarged TH-positive axon terminals of *Atg7* cKO mice. (**A**) Enlarged axon terminals in the striatum of 2-month-old *Atg7* cKO mice were positive for the axon terminal proteins of midbrain DA neurons. Enlarged axon terminals (arrows in green) in *Atg7* cKO mice were stained with VMAT2 (arrows in red). Bars, 10 µm. (**B**, **C**) Enlarged axon terminals in 2-month-old *Atg7* cKO mice are Ub- and p62-negative. Enlarged axon terminals (arrows in green) in the striatum of *Atg7* cKO mice were not stained with the markers for protein inclusions such as Ub (red in ‘B’) and p62 (red in ‘C’), suggesting that they are distinct from the inclusions seen in the cell somas of *Atg7* cKO mice (Figure 1D). Bars, 10 µm. (**D**) Ultrastructural analysis of dopaminergic axon terminals in the striatum of 3-month-old *Atg7* cWT or *Atg7* cKO mice by immunoelectron microscopy with an antibody to TH. Red circles indicate the gold particle-conjugated anti-TH antibody. Bars, 200 nm. (right) Quantification of the size distribution of axon terminals in striatal sections. Each dot (•) represents approximately 1.6% of the total axon terminal number. n = 611 terminals for cWT and 592 terminals for cKO sections.

Grossly enlarged TH-positive dopaminergic axon terminal structures were observed at their striatal target in *Atg7* cKO mice (Figure 1C, E). The axon terminal enlargement was apparent from 2 weeks of age, thus preceding other phenotypes observed in *Atg7* cKO mice, and did not progress with age (Figure 1E). With aging, striatal fiber density of dopaminergic axon terminals declined slowly in *Atg7* cKO mice (Figure 1F), which correlated with the slowly progressive loss of midbrain DA neuron as we have previously reported [16]. The enlarged axon terminals within the striatum were stained positively with an antibody to dopaminergic presynaptic component vesicular monoamine transporter 2 (VMAT2) (Figure 2A). In contrast to the soma inclusions (Figure 1D), the enlarged axon terminals were not stained with antibodies to Ub and p62 (Figure 2B, C).

**Figure 3:**
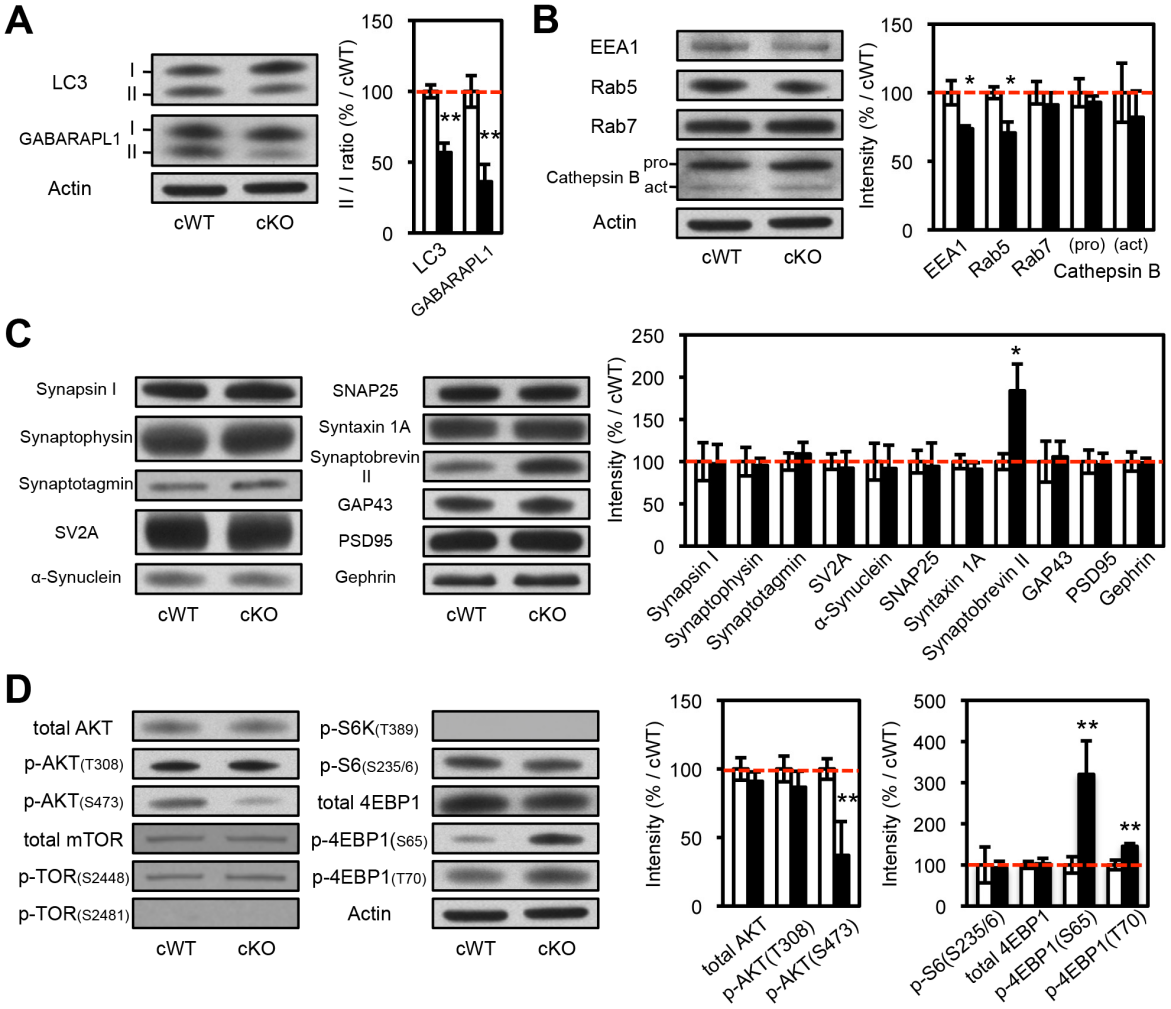
Biochemical analyses of the protein extracts from the striatal synaptosomes of *Atg7* cKO mice. (**A**) Evidence for reduced macroautophagy activity in axon terminals of *Atg7* cKO mice. Conversions of LC3-I and GABARAPL1-I into modified lipidated forms associated with autophagosome formation – termed LC3-II and GABARAPL1-II, respectively – were significantly decreased in striatal synaptosomal preparations from *Atg7* cKO mice (relative to cWT mice); the incomplete reduction likely reflects the presence of non-dopaminergic axon terminals. White, *Atg7* cWT; Black, *Atg7* cKO. n = 5 per group. **, *p*<0.01. (**B**) Moderately reduced accumulation of early endosome markers in striatal synaptosomal preparations from *Atg7* cKO mice. Both EEA1 and Rab5 are significantly decreased in striatal synaptosomal preparations from *Atg7* cKO mice (relative to cWT mice), whereas late endosomal and lysosomal markers, Rab7 and Cathepsin B, unchanged. pro, procathepsin B; act, active mature Cathepsin B. Internal control Actin is same as Figure 3A. White, *Atg7* cWT; Black, *Atg7* cKO. n = 5 per group. *, *p*<0.05. (**C**) Selectively increased accumulation of Synaptobrevin II in striatal synaptosomal preparations from *Atg7* cKO mice. Other synaptic markers were not significantly altered. Internal control Actin is same as Figure 3A. White, *Atg7* cWT; Black, *Atg7* cKO. n = 5 per group. *, *p*<0.05. (**D**) Non-canonical alterations of PI3K/mTOR pathway signaling in synaptosomal preparations from *Atg7* cKO mice. Phosphorylation of AKT at Ser 473 (S473) was decreased in *Atg7* cKO mice, whereas phosphorylation at Thr 308 (T308) unchanged. Phosphorylations of mTOR at Ser 2448 (S2448) or Ser 2481 (S2481) were unchanged in *Atg7* cKO mice. Phosphorylations of 4EBP1 at Ser 65 (S65) and Thr 70 (T70) were increased in the striatal synaptosomes of *Atg7* cKO mice, whereas phosphorylations of S6 (T389) and S6K (S235/236) were unchanged. Internal control Actin is same as Figure 3A. n = 5 per group. **, *p*<0.01.

Biochemical analysis of striatal synaptosomal preparations from 2-month-old *Atg7* cKO mice and their littermates revealed predominantly unchanged levels of pre- and post-synaptic proteins including Synapsin I, Synaptophysin, Synaptotagmin, Synaptic vesicle protein 2A (SV2A), α-Synuclein, Synaptosomal-associated protein 25 kDa (SNAP25), Syntaxin 1A, Growth associated protein 43 (GAP43), Postsynaptic density protein 95 (PSD95), and Gephrin (Figure 3C). Levels of early endosomal compartment markers present at presynaptic terminals, including early endosome antigen-1 (EEA1) and Rab5, were reduced, whereas late endosomal/lysosomal markers including Rab7 and Cathepsin B appeared unchanged (Figure 3B). An additional presynaptic marker protein, Synaptobrevin II, appeared increased in accumulation in *Atg7* cKO synaptosomal preparations (Figure 3C). Thus, macroautophagy deficiency in midbrain DA neurons leads to axonal terminal enlargement associated with modest alternations in the accumulation of presynaptic regulatory proteins.

Consistent with these findings, immunoelectron microscopy for DA neuron marker, TH, showed significant enlargement of Atg7 deficient dopaminergic axon terminals but otherwise normal appearing morphology, including presynaptic terminals, synaptic vesicles, and mitochondria (Figure 2D). Furthermore, no inclusions or membrane swirls were apparent, in contrast to those described within dystrophic neuronal terminals in Atg7 deficient Purkinje neurons [5]. Thus, the morphology phenotype does not appear to be a consequence of the accumulation of unfolded protein in the context of aberrant degradation, nor represent dystrophic changes as described in other neuronal populations deficient in Atg7 [5].

**Figure 4:**
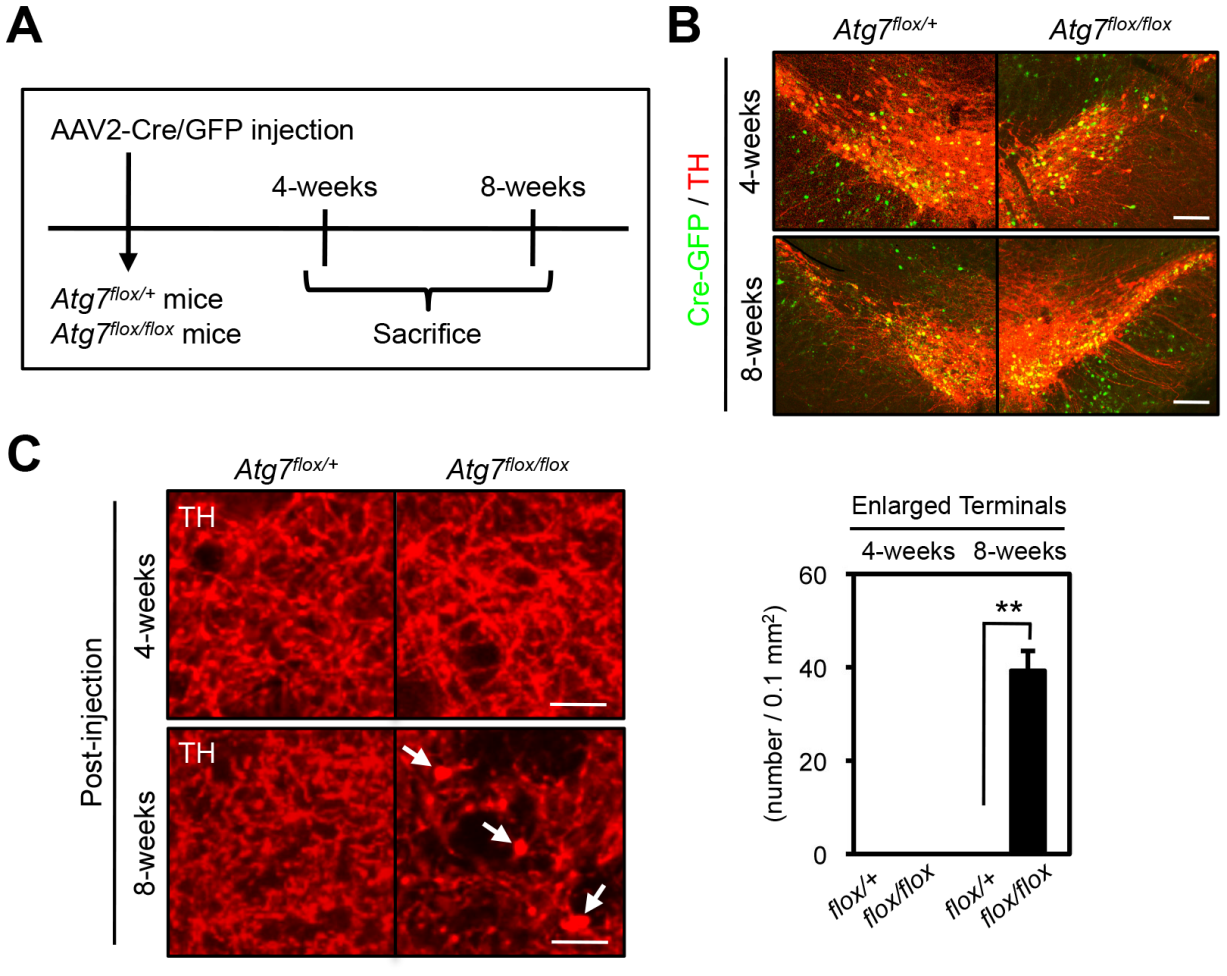
Atg7 regulates morphological plasticity of mature dopaminergic axon terminals. (**A**) Scheme of AAV2-Cre/GFP viral transduction of adult *Atg7^flox/flox^* substantia nigra. Eight-week-old *Atg7^flox/+^* and *Atg7^flox/flox^* mice were stereotactically injected with AAV2-Cre/GFP viral solution, and sacrificed 4- or 8-weeks later. (**B**) AAV2-Cre/GFP viral transduction of substantia nigra led to prominent GFP fluorescence (green) in a majority of TH-positive DA neurons (red) at 4- or 8-weeks after the injection; no GFP fluorescence was seen in untransduced animals (data not shown). (**C**) Transduction of Cre/GFP virus into adult *Atg7^flox/flox^* substantia nigra reproduced the enlarged axon terminal phenotype seen in *Atg7* cKO mice. At 8-weeks after injection, enlarged TH-positive axon terminals (arrows) were seen in the striatum of *Atg7^flox/flox^* mice injected with AAV2-Cre/GFP viral solution. No enlarged axon terminals were seen in the striatum of *Atg7^flox/+^* mice with AAV2-Cre/GFP virus or *Atg7^flox/flox^* mice with control AAV2-GFP virus lacking Cre. Bars, 20 µm. (right) Quantification of the density of enlarged axon terminals in mice injected with AAV2-Cre/GFP virus. TH-positive axon terminal enlargement in *Atg7^flox/flox^* mouse striatum was seen at 8-weeks after the AAV2-Cre/GFP virus injection. White, *Atg7^flox/+^*; Black, *Atg7^flox/flox^*. n = 3 per group. **, *p*<0.01.

We sought to further address whether the axon terminal morphological change in the context of Atg7 deficiency is a consequence of altered development or altered maintenance of mature axon terminals. Thus, adult 2-month-old *Atg7^flox/flox^* mice, which remain intact for Atg7 expression, were stereotaxically injected with adeno-associated virus-2 (AAV2) that harbors Cre/green fluorescence protein (GFP) or GFP control into the ventral midbrain unilaterally (Figure 4A), effectively transducing a large fraction of TH-positive DA neurons (Figure 4B). Analysis of these mice 8 weeks after viral transduction revealed the dramatic enlargement of dopaminergic axon terminals within the striatum only of the AAV2-Cre/GFP virus transduced *Atg7^flox/flox^* mice (Figure 4C), consistent with the phenotype in *Atg7* cKO mice. Thus, these studies confirm a role for Atg7 in the morphological plasticity of mature dopaminergic axon terminals.

**Figure 5:**
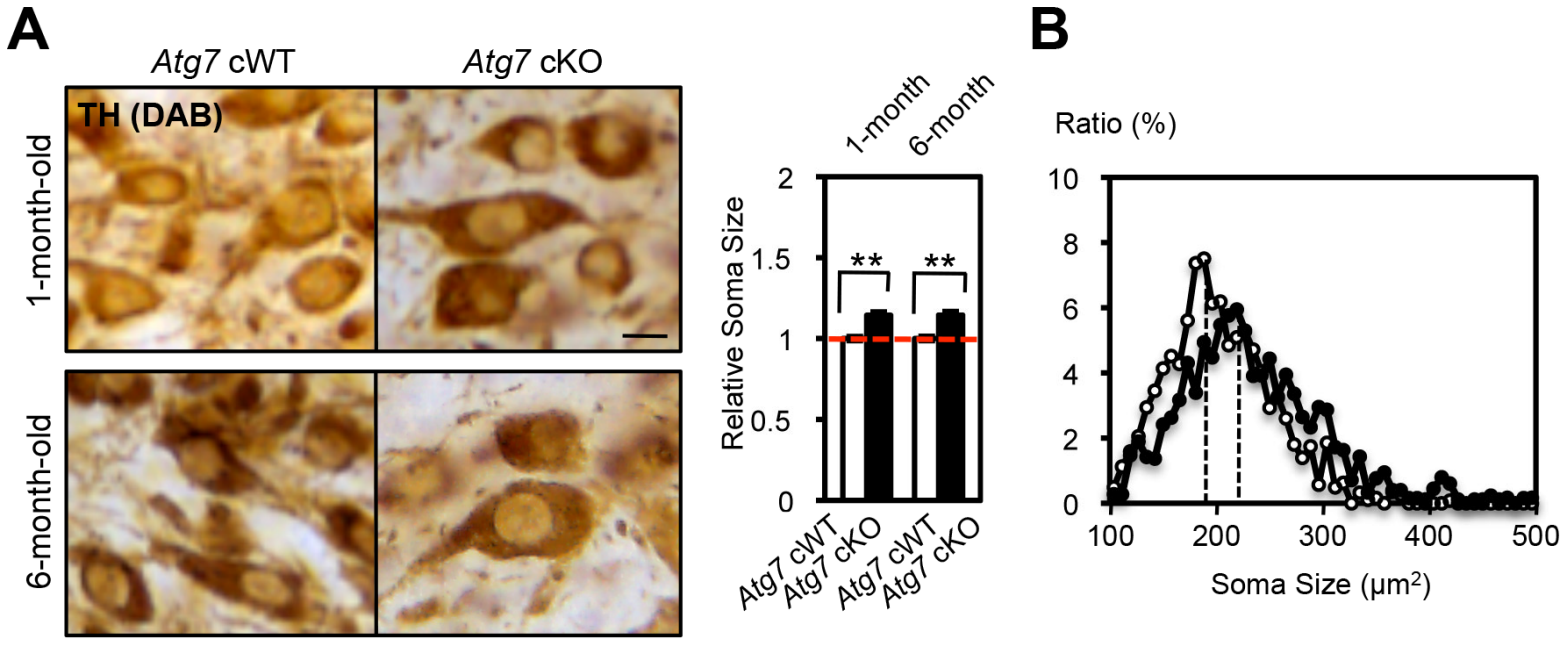
Enlarged soma size in TH-positive DA neurons of *Atg7* cKO mice. (**A**) Enlarged cell soma of TH-positive DA neurons in *Atg7* cKO mice. The soma area (brown in pictures) of nigral TH-positive DA neurons in *Atg7* cKO mice (black in graph) was approximately 15% larger than that in *Atg7* cWT mice (white in graph), as quantified using Image-J software (Image J, Bethesda, MD) and presented relative to the area of the *Atg7* cWT group. Bars, 10 µm. (*Atg7* cWT mice = 1.0); n = 194 to 377 TH-positive DA neurons per group. **, *p*<0.01. (**B**) The distribution of cell soma size of TH-positive DA neurons in *Atg7* cWT and *Atg7* cKO mice. The soma size of TH-positive DA neurons in *Atg7* cKO mice (black circle) was on average approximately 15% larger than that in *Atg7* cWT mice (white circle).

In addition to the axon terminal enlargement, at time points as early as 1-month of age, morphometric analysis of TH-positive cell soma in *Atg7* cKO mice (or *Atg7* cWT mice) revealed a significant albeit minor increase in soma size (15% increase, Figure 5A, B). This phenotype appeared similar in older animals (and thus not age-dependent; Figure 5A), and thus was not correlated with the neurodegenerative phenotype seen with aging.

As axon terminal enlargement appeared non-progressive and was not associated with intracellular inclusions or other apparent pathological changes, this was unlikely to be secondary to the late-onset progressive degeneration seen in midbrain DA neurons of *Atg7* cKO mice [19], [20]. We note that *in vitro* studies using primary midbrain cultures prepared from *Atg7* cKO or littermate cWT embryos further support this interpretation. TH-positive DA neurons in *Atg7* cKO cultures at day 5 *in vitro* (5th DIV) displayed increased total neurite length (Figure S1B). Importantly, TH-positive DA neuron number and appearance was otherwise not altered in the *Atg7* cKO primary cultures (Figure S1B), and thus this early phenotype is not likely to reflect degeneration.

### mTOR pathway modification in Atg7 deficient dopaminergic axon terminals

The mTOR kinase signaling pathway is a key regulator of mammalian cell size [8], [9], [21]–[23]. As mTOR is also a major negative regulator of macroautophagy [24], we hypothesized that Atg7 may function in a common pathway with mTOR, or parallel to mTOR, in the context of dopaminergic axon terminal size regulation. To this end, we evaluated mTOR pathway activation within dopaminergic axon terminal projections in the striatum of *Atg7* cKO or cWT mice. Crude striatal synaptosomal protein fractions were prepared and analyzed by Western blotting. As expected, *Atg7* cKO synaptosomes displayed evidence of reduced macroautophagy, as the lipidation of LC3 (as well as of the related autophagosome marker protein GABARAPL1) was reduced (Figure 3A). However, the pattern of mTOR pathway component modification was not consistent with canonical activation of the PI3K/mTOR pathway. Phosphorylation of eukaryotic translation initiation factor 4E binding protein 1 (4EBP1), a downstream target and effector of mTOR signaling [25], was significantly increased at Ser 65 and Thr 70 in *Atg7* cKO mice (Figure 3D), but phosphorylation of other typical downstream targets of mTOR – ribosomal protein S6 kinase (S6K) (Thr 389) and S6 (Ser 235/236) – appeared unchanged (Figure 3D). Furthermore, canonical PI3K pathway-associated modifications of mTOR kinase – in terms of the accumulation of phospho-mTOR (Ser 2448 and Ser 2481; Figure 3D, 6C) – or of the upstream PI3K pathway component AKT kinase (Ser 473), were not evident in *Atg7* cKO striatum (Figure 3D). Taken together, these findings argue against a simple model whereby Atg7 deficiency may modify axonal process morphology through the modification of PI3K/mTOR downstream pathway (Figure S3A). A caveat to the interpretation of mTOR pathway modification using striatal synaptosome extracts is that non- dopaminergic axon terminals are also present; however, Atg7 deletion was restricted to midbrain DA neurons.

**Figure 6:**
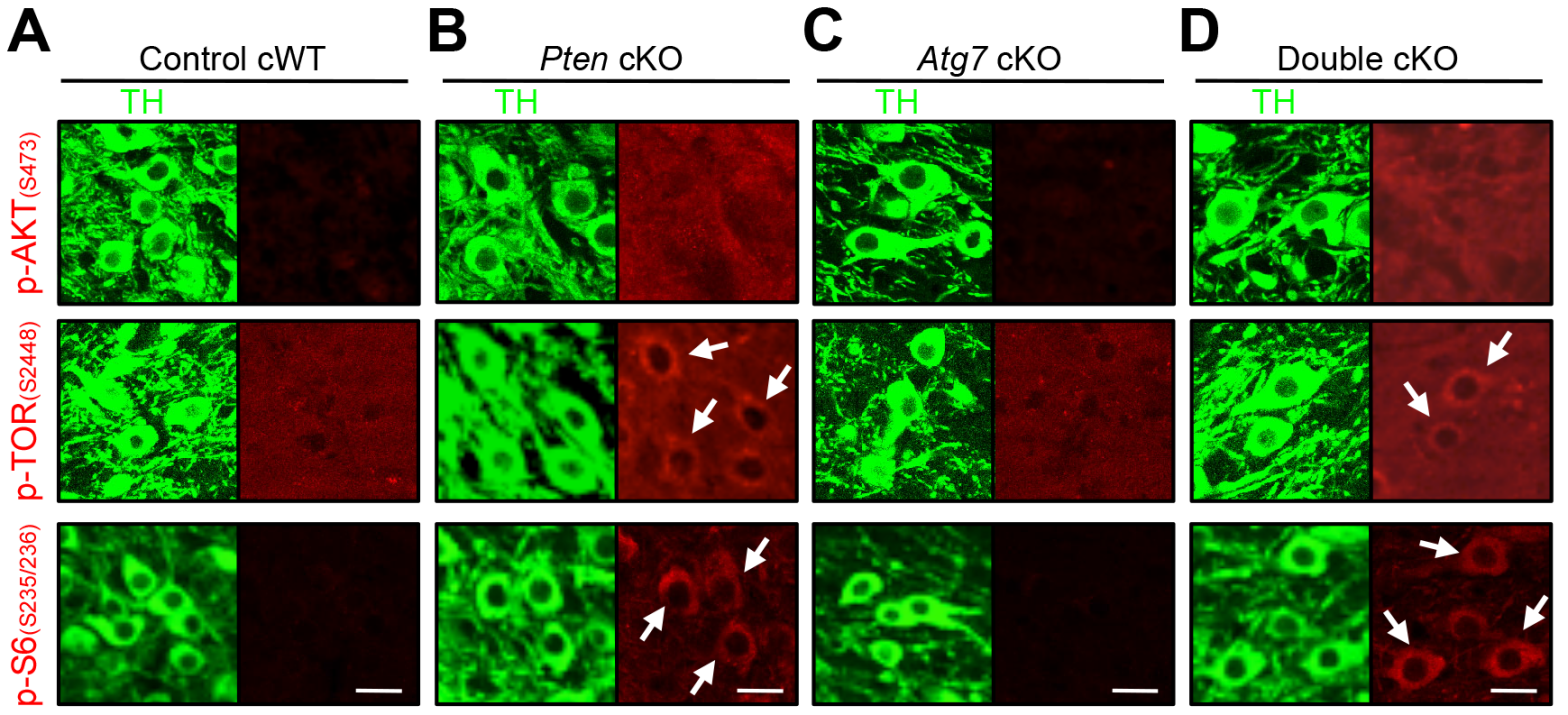
Characterization of PI3K/mTOR pathways in TH-positive DA neurons of *Atg7* and/or *Pten* cKO mice. PI3K/mTOR pathway signaling, in terms of accumulation of phospho-AKT (S473), phospho-TOR (S2448), and phospho-S6 (S235/236) as indicated (in red) were unchanged in the TH-positive (green) midbrain DA neurons of *Atg7* cKO mice (C), whereas these markers were increased in TH-positive DA neurons of *Pten* cKO and *Atg7/Pten* double cKO mice (arrows in B and D). (**A**) Control cWT mice, (**B**) *Pten* cKO mice, (**C**) *Atg7* cKO mice, and (**D**) *Atg7/Pten* double cKO mice. Scale bars, 10 µm.

### Pten deficient midbrain DA neurons display enlarged cell soma but unaltered axon terminals

**Figure 7:**
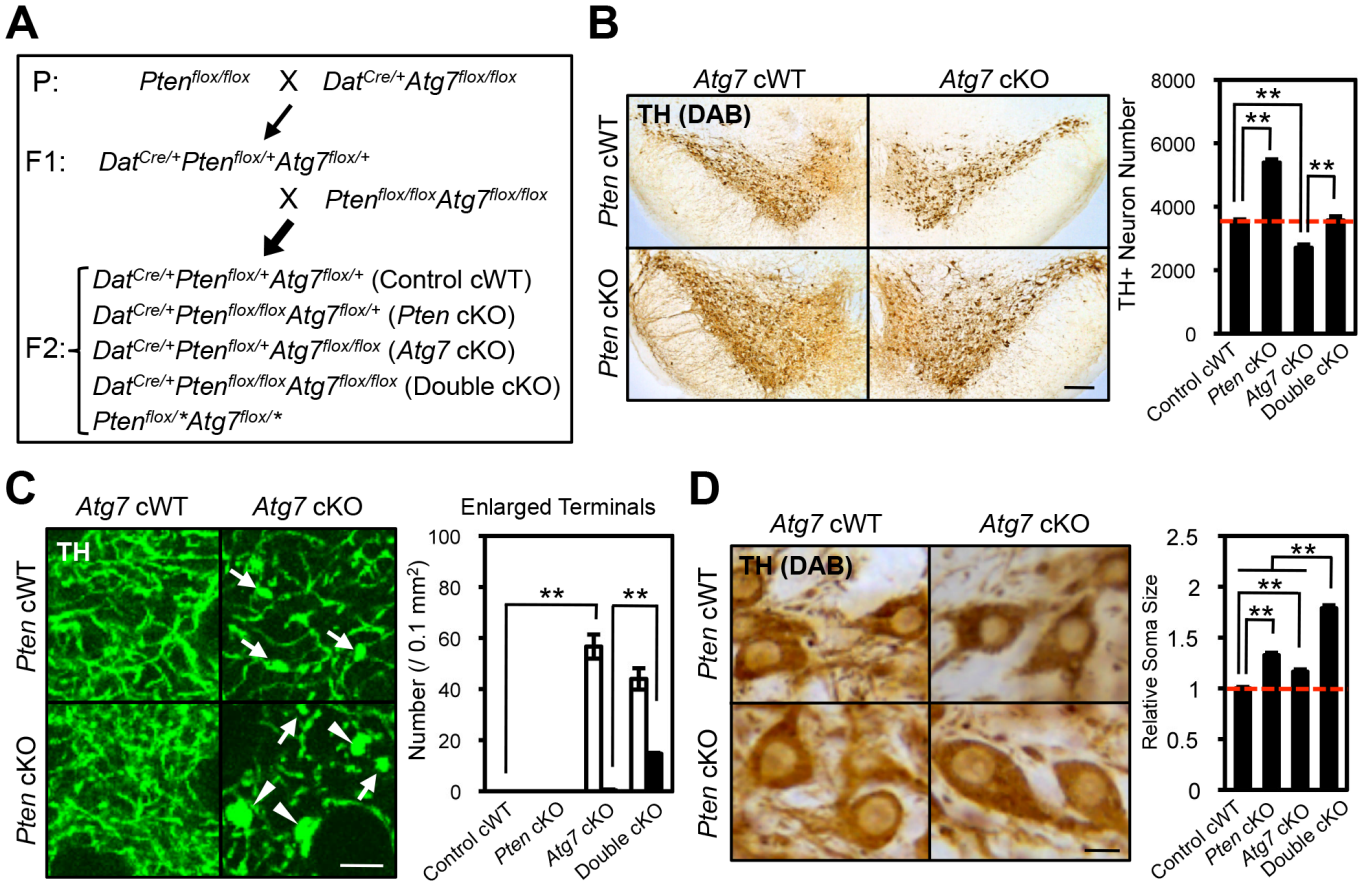
Atg7 and Pten double deficiency synergistically increases axon terminal size in midbrain DA neurons. (**A**) Schema of mouse mating to obtain Atg7 and Pten double deficient mice. *Dat^Cre/+^* background animals were used for the analyses. Animals lacking *Cre* (Pten^flox/^*Atg7^flox/^*) were not used in this study. Asterisk indicates ‘+’ or ‘flox’. (**B**) Neurodegeneration in *Atg7* cKO mice was rescued by secondary deletion of Pten. Secondary Pten deletion (*Atg7/Pten* double cKO) suppressed the loss of TH-positive DA neurons in the substantia nigra of 2-month-old *Atg7* cKO mice. Representative TH-stained midbrain sections are presented. Bar, 250 µm. (right) Quantification of TH-positive DA neuron number in the substantia nigra of *Atg7/Pten* double cKO mice. n = 4 per genotype. **, *p*<0.01. (**C**) The enlarged axon terminal phenotype of *Atg7* cKO mice was greatly enhanced in *Atg7/Pten* double cKO mice, whereas Pten deficiency alone (*Pten* cKO) did not significantly change the axon terminal size. (left) Giant (arrowheads, >9.8 µm^2^) and moderately enlarged (arrows, 4.4∼9.8 µm^2^) axon terminals were seen in the striatum of *Atg7/Pten* double cKO mice, whereas only moderately enlarged axon terminals (arrows) were seen in *Atg7* cKO mice and no enlarged axon terminals were seen in *Pten* cKO mice. Bars, 20 µm. (right) Quantification of enlarged axon terminal distribution. Black bar, giant terminals (>9.8 µm^2^); white bar, moderately enlarged terminals (4.4∼9.8 µm^2^). n = 6 per genotype. **, *p*<0.01. (**D**) The soma of TH-positive DA neurons in *Atg7/Pten* double cKO mice were dramatically enlarged (79% increase versus control cWT) relative to *Atg7* cKO mice (15% increase versus control cWT) and *Pten* cKO mice (32% increase versus control cWT). (left) Representative sections stained with anti-TH antibody. Bars, 10 µm. (right) Quantification of the average cell size, presented as a fraction of DA neuron soma size in control cWT mice. n = 290∼417 TH-positive DA neurons per genotype. **, *p*<0.01.

We next sought to more directly compare the roles of PI3K/mTOR pathway modification and macroautophagy in the context of dopaminergic axon terminal size. To this end, we generated mice deficient in Pten specifically within midbrain DA neurons (*Dat^Cre/+^Pten^flox/flox^* [*Pten* cKO]). As expected, *Pten* cKO midbrain sections displayed canonical activation of PI3K/mTOR pathway, quantified in terms of accumulation of phospho-AKT at Ser 473, phospho-mTOR at Ser 2448 and phospho-S6 at Ser 235/236 (Figure 6A, B) [14], [15]. In contrast with *Atg7* cKO mice, *Pten* cKO mice displayed no significant alteration in axon terminal size in the striatum (Figure 7C) [14]. Nonetheless, *Pten* cKO mice displayed robustly increased soma size of DA neurons (30% increase, Figure 7D), consistent with two prior studies of Pten deficiency [14], [15], and which was much more profound than the modest soma alteration in the context of Atg7 deficiency (Figure 7D). Thus, although both Pten deficiency and Atg7 deficiency modify cell morphology, the phenotypes are distinct, and thus the effect of Atg7 deficiency cannot simply reflect altered PI3K/mTOR pathway activation alone.

### Synergistic enlargement of axon terminal size in Atg7 and Pten double deficient DA neurons

**Figure 8:**
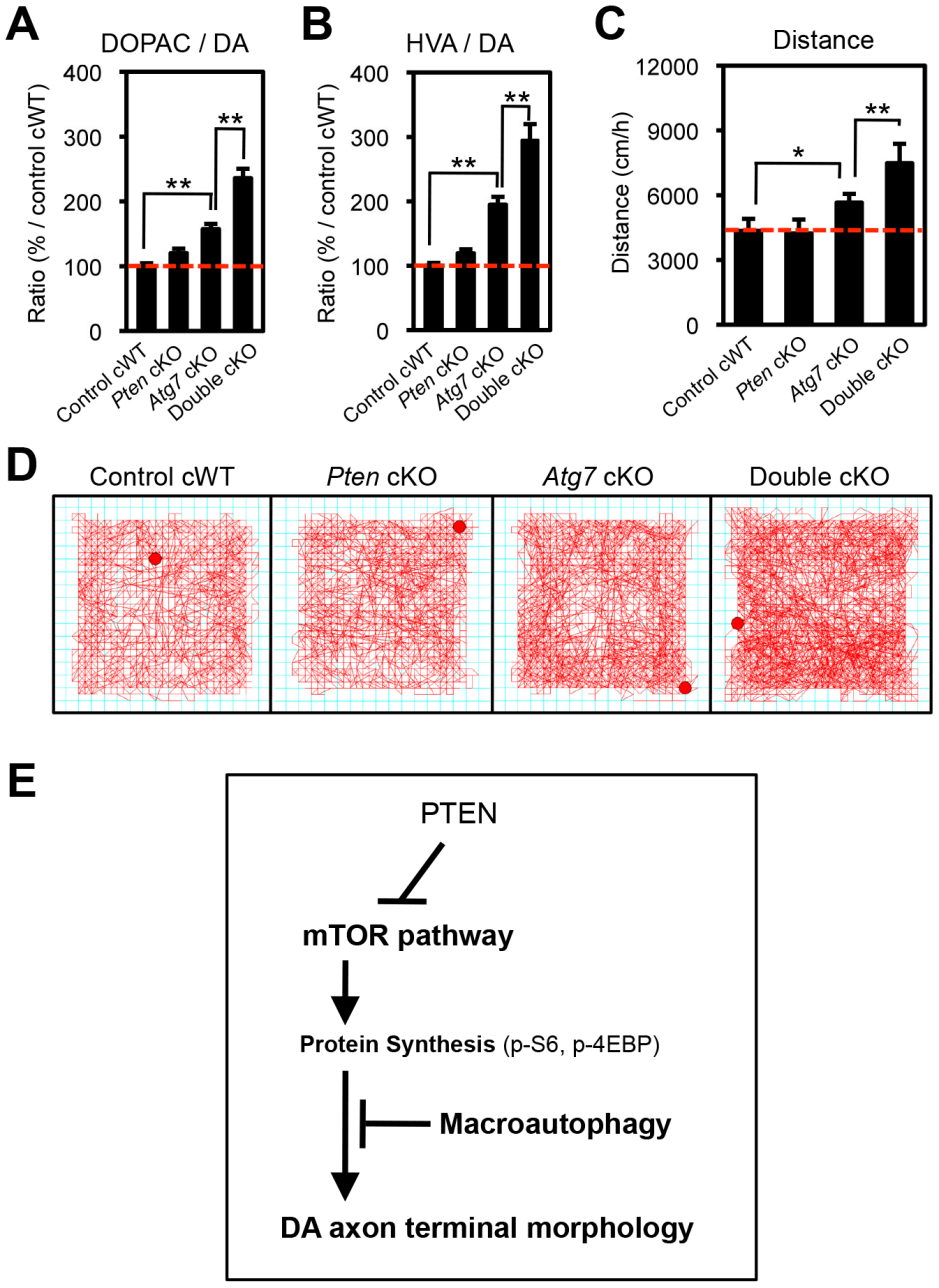
Atg7 and Pten double deficiency synergistically increases DA turnover and DA-associated behaviors. (**A**, **B**) DA turnover, quantified as the ratio of the DA metabolites DOPAC or HVA to DA (DOPAC/DA [A] or HVA/DA [B]) was synergistically increased in *Atg7/Pten* double cKO mice. The concentrations of DA, DOPAC, and HVA are shown in Figures S2A–C. n = 10∼12 per genotype. **, *p*<0.01. (**C**, **D**) Basal locomotor activity was synergistically increased in *Atg7/Pten* double cKO mice. Traces (‘D’) display total distance traveled over a 30-min period in an open field environment. Walking velocity was unchanged (Figure S2D). n = 10∼12 per genotype. *, *p*<0.05; **, *p*<0.01. (**E**) In midbrain DA neurons at baseline, Atg7-mediated macroautophagy inhibits the enlargement of dopaminergic axon terminals, in part by masking the impact of the PTEN/PI3K/mTOR pathway. Also see Figure S3.

To further consider the genetic relationship of *Atg7* and *Pten* in the context of axon morphology, we generated double mutant mice lacking both *Atg7* and *Pten* specifically in midbrain DA neurons (*Atg7*/*Pten* double cKO), and compared these to single cKO mice (either *Atg7* cKO or *Pten* cKO alone) as well as to control cWT mice (Figure 7A). PI3K/mTOR pathway activation was apparent in the *Atg7*/*Pten* double cKO mice, as expected, with accumulation of phospho-AKT at Ser 473, phospho-mTOR at Ser 2448, and phsopho-S6 at Ser 235/236 in midbrain DA neurons (Figure 6D) comparable to that observed in the *Pten* single cKO mice (Figure 6B). Surprisingly, dopaminergic axon terminals were dramatically larger in the double cKO mice than those in *Atg7* cKO mice or control animals (Figure 7C, giant axon terminals). These giant axon terminals in *Atg7/Pten* double cKO mice were positive to VMAT2, but negative to Ub and p62 (data not shown). *Atg7/Pten* double cKO mice also showed mildly potentiated soma enlargement (79% increase, Figure 7D) relative to either single cKO mice. Thus, PI3K/mTOR pathway activation alone is not sufficient to modify dopaminergic axon terminal size, but its impact is unmasked in the context of Atg7 deficiency (Figure 8E). A further consequence of Pten deficiency in the context of Atg7 loss is that subsequent progressive midbrain DA neuron degeneration, as seen in 2-month-old *Atg7* cKO mice and thereafter, is suppressed (Figure 7B). As such ‘rescue’ of neurodegeneration in *Atg7*/*Pten* double cKO mice failed to prevent the enlarged axon terminal phenotype (but instead actually enhanced the enlargement; Figure 7C), this further validates the notion that axon terminal enlargement in *Atg7* cKO mice is not a consequence of neurodegeneration.

### Synergistic impact of Atg7 and Pten deficiency on DA metabolism and DA-associated locomotion

The studies above detail a synergistic role for *Atg7* and *Pten* in the regulation of DA neuron morphology. We sought to expand the synergistic effects to functional changes in *Atg7* cKO mice. Striatal DA accumulation and its metabolites, 3,4-dihydroxyphenylacetic acid (DOPAC) and homovanillic acid (HVA), were quantified in 3-month-old single and double mutant mice (Figure S2A–C). DA turnover (DOPAC/DA and HVA/DA) in the striatum, which is a reflection of dopaminergic axon terminal activity, was increased in *Atg7* cKO mice (relative to control cWT mice; Figure 8A, B). This phenotype was further enhanced in *Atg7/Pten* double cKO mice, whereas *Pten* cKO alone appeared normal (Figure 8A, B), mirroring the morphological findings. The absolute level of DA accumulation, in contrast to DA turnover, was significantly reduced in *Atg7* cKO mice (Figure S2A), which may reflect cell loss at this age. However, this reduction in DA levels was not ‘rescued’ by PTEN loss (Figure S2A).

Given the altered DA accumulation in *Atg7* cKO mice, as well as prior studies demonstrating physiological changes in these animals [26], we sought to identify possible behavioral correlates. To this end, basal locomotor activity was quantified in an open field chamber that was novel to the animals over a 30-min period. Ambulatory distance travelled was increased in *Atg7* cKO mice (relative to control cWT mice; Figure 8C, D). This phenotype was further enhanced in *Atg7/Pten* double cKO mice (Figure 8C, D), whereas *Pten* single cKO mice behavior did not appear significantly altered (Figure 8C, D) [14]. Other activity parameters, including jump counts and vertical activity, appeared similarly altered in the *Atg7* cKO mice and *Atg7/Pten* double cKO mice (Figure S2E, F). In contrast, velocity of ambulation was not altered (Figure S2D). Thus, *Atg7* single cKO and *Atg7/Pten* double cKO mice displayed alterations in motor behavior that correlated with their cellular changes and alterations in DA turnover.

## Discussion

The PTEN/PI3K/mTOR signaling pathway plays a central role in the regulation of neuronal morphology and size in developing vertebrate and invertebrate species [8], [9], [21], [22]. However, a number of studies have provided evidence that in the context of the mature mammalian CNS, as well as within certain subcellular compartments of neurons such as at axonal processes, the impact of the PTEN//PI3K/mTOR pathway on size can be highly regulated. For instance, although deletion of PTEN in post-mitotic DA neurons leads to enlarged soma (Figure 7D) [14], [15], dopaminergic axon terminals are not altered (Figure 7C) [14], [15]. In contrast to these observations, PTEN deletion at earlier developmental stages or in other neuronal types, such as in the progenitors of dentate gyrus granule neurons [10], [11], leads to prominent enlargement of axon terminals as well as soma. Thus, mechanisms that regulate the impact of the PI3K/mTOR pathway on neuronal morphology are of particular interest. Here we show that deletion of the essential macroautophagy component Atg7 unmasks the impact of Pten deletion on dopaminergic axon terminal size. These data implicate macroautophagy as a negative regulator of PTEN/PI3K/mTOR pathway regulation of neuronal morphology (Figure 8E).

Although the precise mechanism by which Pten deletion or PI3K pathway activation leads ultimately to cellular hypertrophy remains unclear, multiple studies in Drosophila [27] and mice [23], [28], [29] have implicated activation of the ribosomal protein S6 by a family of related kinases that include S6K1 and S6K2, leading to increased protein translation [30]. As neither S6 phosphorylation nor activation of S6K appeared modified in the context of Atg7 deficiency (Figure 3D), the axonal process enlargement seen with macroautophagy deficiency is likely to be through a distinct mechanism. Our genetic studies strongly validate this notion, as PI3K/mTOR pathway activation (by means of PTEN deficiency) and Atg7 deficiency act cooperatively and synergistically in modifying axon terminal morphology. We favor an interpretation whereby at the axonal terminus, macroautophagy-mediated protein degradation is typically able to overcome the increased protein production in the context of mTOR pathway activation, and thus macroautophagy typically suppresses the impact of Pten deletion on dopaminergic axon terminal size. However, in the context of defective macroautophagy with Atg7 deficiency, the impact of Pten deletion is unmasked (Figure 8E). In addition to suppressing PTEN/PI3K/mTOR pathway-mediated regulation, macroautophagy likely plays additional roles in determining dopaminergic axon terminal size and function, as the impact of Atg7 deficiency was observed regardless of Pten deficiency (Figure 7C).

Our data argue against an alternative model whereby mTOR pathway activation dictates dopaminergic axon terminal morphology through the downstream inhibition of macroautophagy (rather than through downstream effects on the translation machinery) (Figure S3A), as has been suggested based on the recent *in vitro* analyses of acutely prepared striatal slice preparations treated with the mTOR inhibitor rapamycin [26]. The impact of the PTEN/PI3K/mTOR pathway on mature dopaminergic axon terminal morphology in our present study was apparent in the complete absence of macroautophagy (when comparing *Atg7* single mutant mice with *Atg7/Pten* double mutant mice), and thus the mechanism of action cannot be explained simply by alterations in macroautophagy activity. It remains possible that alterations in macroautophagy activity play some role downstream of mTOR pathway activation in the context of more acute physiological changes at axon terminals [26]. Nonetheless, our findings support a distinct model whereby macroautophagy plays a key role in suppressing the impact of mTOR pathway activation in the context of mature dopaminergic axon terminals (Figure 8E).

Previous studies have reported that midbrain DA neuron-specific Atg7 loss [19], as well as loss of Atg7 in other neuronal classes, leads to enlarged but dystrophic axons [5], [6]. These findings have generally been interpreted as secondary effects of the accumulation of pathological inclusions (‘engorgement’). However, in our analyses of Atg7 deficient midbrain DA neurons, dopaminergic axon terminal enlargement preceded degeneration, appeared non-progressive (Figure 1E), and was not associated with protein aggregates (Figure 2B, C). Furthermore, this phenotype was enhanced – rather than suppressed – in the context of additional Pten deficiency (*Atg7/Pten* double deficient animals) (Figure 7C), although such additional Pten deficiency effectively suppressed the late-onset degeneration phenotype of the Atg7 deficient midbrain DA neurons (Figure 7B) [16].

Prior studies in invertebrate species have suggested a role for macroautophagy in axon terminal morphology. For instance, at the *Drosophila* neuromuscular junction (NMJ), macroautophagy has been reported to promote synapse development through selective degradation of the *Highwire* ubiquitin ligase [31]. Mutations in C. elegans *unc-51* –a macroautophagy regulator– lead to developmental axonal defects [32]. There is also precedent of a role for macroautophagy in cell size homeostasis: induction of macroautophagy by Atg1 leads to the reduced *Drosophila* fat body cell size in TOR signaling-dependent manner [33]. In mammalian models, loss of macroautophagy-associated proteins other than Atg7 has similarly been implicated in axonal morphology, although the mechanism has remained unclear. Mammalian *Ulk1/2*, orthologues of Unc51, have been reported to regulate axonal outgrowth [34]–[36]. Taken together, we speculate that regulation of axon terminal size by macroautophagy may play an important role in structural plasticity at mature adult axon terminals. Extrinsic cues such as glial derived neurotrophic factor (GDNF) impact dopaminergic axon terminal structures and modify signaling through the PI3K pathway [37], [38]; it will be of interest to pursue the role of macroautophagy in such changes, which have been implicated clinically in pathological movements associated with experimental therapeutics for Parkinson's disease [39]–[42].

Future studies will seek to identify specific molecular components that may mediate dopaminergic axon terminal enlargement in the context of defective macroautophagy. Our initial screen of known axon terminal proteins revealed the increased accumulation of Synaptobrevin II in the context of Atg7 deficient dopaminergic axon terminal preparations (Figure 3C). In addition to proteins, macroautophagy also plays a role in cell membrane regulation [43], and this may also impact axon terminal morphology. Finally, it is interesting to note that axon terminal morphology, DA neurons, and the PI3K pathway [44]–[47] have all been implicated in the etiology of autism spectrum disorders (ASD), which are characterized by cognitive difficulties and can be associated with hyperactivity 48,49. Furthermore, recent studies have suggested a role for alterations in the protein degradation machinery in ASD [50]–[52]. We thus speculate a role for macroautophagy regulation of axon terminal morphology in the context of brain disorders.

## Materials and Methods

### Animal

*Dat^Cre/+^* mice, *Atg7^flox/flox^* mice, and *Pten^flox/flox^* mice were generated previously [17], [18], [53]. All animals were maintained in the animal facility of Columbia University Medical Center. All of the experimental protocols were approved by the Institutional Animal Care and Use Committees. All mice we used were *Dat^Cre/+^* background, as *Dat* heterozygous KO mice show some defects in their behavior and physiology [54].

### Histology

Mice were perfused in 4% paraformaldehyde and 50 µm coronal sections were made by a vibratome. The antibodies used here were listed in Text S1.

### Electron microscopy

Electron microscopic analysis was according to the previous paper [55] and Immunogold incubation protocol for general application (Electron Microscopy Sciences, Hatfield, PA).

### Cell size determination

After the TH staining of midbrain sections by DAB, pictures were taken at 400× magnification. The size of TH-neuron was measured manually by Image-J (NIH) [56]. More than 200 TH-neurons from 4 mice were analyzed per group.

### Western blotting

Preparation of the striatal synaptosomal fractions was according to the previous paper [57]. The antibodies used here were listed in Text S1.

### High performance liquid chromatography (HPLC)

The striatal tissues were used for HPLC analysis. Concentrations of DA and its metabolites were measured according to the previous paper [58].

### Statistical analysis

All of the comparisons were made with Mann-Whitney U-test (for 2 samples) or non-repeated measures ANOVA (for multiple samples). The values are expressed as the means ± SEM. A *p* value less than 0.05 is considered significant.

## Supporting Information

Figure S1 Characterization of enlarged axon terminals of *Atg7* cKO mice. (A) Decreased macroautophagy activity in midbrain extracts from *Atg7* cKO mice. The conversion of LC3-I to LC3-II was reduced in 2-month-old *Atg7* cKO mice. n = 5 per genotype. *, *p*<0.05. (B) Increased neurite length of *Atg7* cKO midbrain TH-positive primary neurons. Total neurite length was significantly increased in *Atg7* cKO primary neurons (bottom right), whereas the total number of TH-positive neurons per well unchanged. Primary midbrain neuron cultures were prepared from 3 embryos per genotype. **, *p*<0.01.
(TIF)

Figure S2 Characterization of *Atg7/Pten* double cKO mice. (A–C) Concentrations of DA, DOPAC, and HVA in the striatum tissues of *Atg7/Pten* double cKO mice. (A) DA. (B) DOPAC. (C) HVA. n = 10∼12 mice per genotype. **, *p*<0.01. (D–F) Quantifications of the parameters in open field test. (D) Walking velocity. (E) Jump counts. (F) Vertical behavior counts. n = 10∼12 per genotype. *, *p*<0.05.
(TIF)

Figure S3 Models for the role of macroautophagy in midbrain DA neuron. Two distinct models for the role of macroautophagy in regulating DA axon terminal morphology and function. (A) In the linear model proposed by the prior study [26], the primary action of mTOR on DA axon morphology is directly through the inhibition of macroautophagy. (B) In our sculptural model, macroautophagy plays a key role in suppressing the action of mTOR signaling at DA axon terminal morphology.
(TIF)

Text S1 Supporting materials and methods.
(DOC)
