# Supplementary figures and images for: Coordinate Regulation of Mature Dopaminergic Axon Morphology by Macroautophagy and the PTEN Signaling Pathway
Source: PLoS Genet. 2013 Oct 3;9(10):e1003845. doi: 10.1371/journal.pgen.1003845 (PMC3789823; doi:10.1371/journal.pgen.1003845)

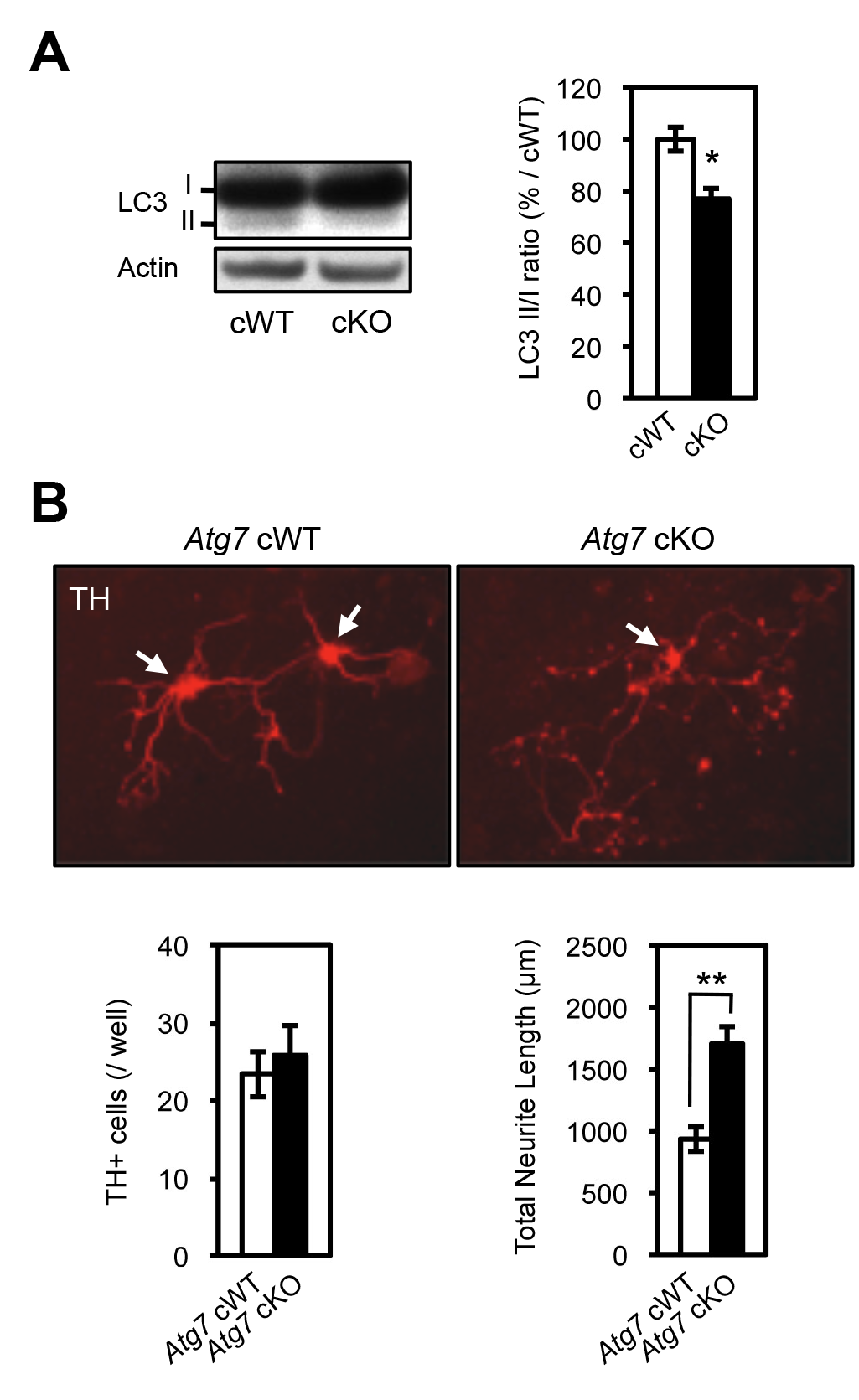

Supplement: Figure S1 — Characterization of enlarged axon terminals of Atg7 cKO mice. (A) Decreased macroautophagy activity in midbrain extracts from Atg7 cKO mice. The conversion of LC3-I to LC3-II was reduced in 2-month-old Atg7 cKO mice. n = 5 per genotype. *, p<0.05. (B) Increased neurite length of Atg7 cKO midbrain TH-positive primary neurons. Total neurite length was significantly increased in Atg7 cKO primary neurons (bottom right), whereas the total number of TH-positive neurons per well unchanged. Primary midbrain neuron cultures were prepared from 3 embryos per genotype. **, p<0.01. (TIF) [file pgen.1003845.s001.tif]

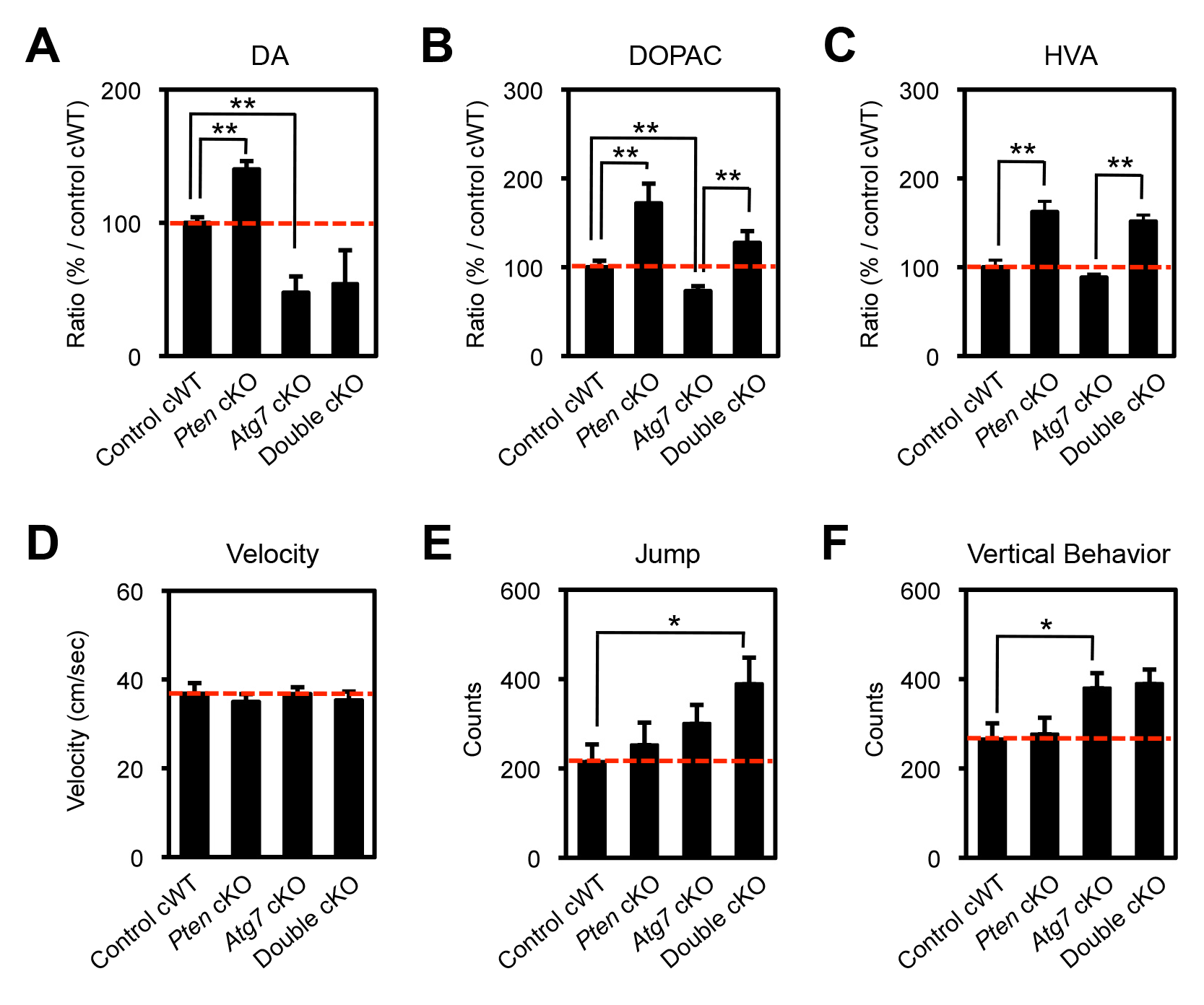

Supplement: Figure S2 — Characterization of Atg7/Pten double cKO mice. (A–C) Concentrations of DA, DOPAC, and HVA in the striatum tissues of Atg7/Pten double cKO mice. (A) DA. (B) DOPAC. (C) HVA. n = 10∼12 mice per genotype. **, p<0.01. (D–F) Quantifications of the parameters in open field test. (D) Walking velocity. (E) Jump counts. (F) Vertical behavior counts. n = 10∼12 per genotype. *, p<0.05. (TIF) [file pgen.1003845.s002.tif]

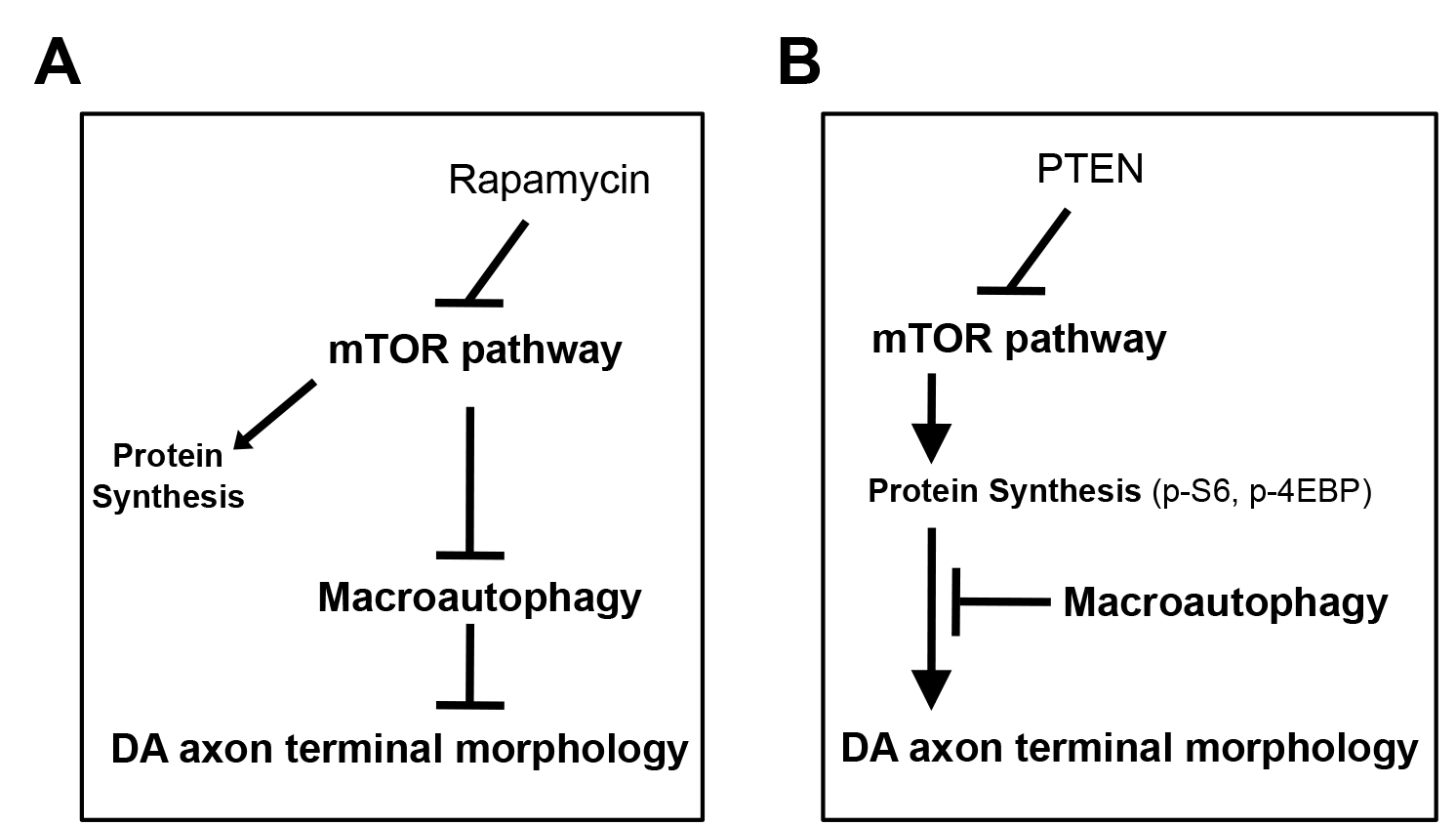

Supplement: Figure S3 — Models for the role of macroautophagy in midbrain DA neuron. Two distinct models for the role of macroautophagy in regulating DA axon terminal morphology and function. (A) In the linear model proposed by the prior study [26], the primary action of mTOR on DA axon morphology is directly through the inhibition of macroautophagy. (B) In our sculptural model, macroautophagy plays a key role in suppressing the action of mTOR signaling at DA axon terminal morphology. (TIF) [file pgen.1003845.s003.tif]
