# Supplementary material for: Coordinate Regulation of Mature Dopaminergic Axon Morphology by Macroautophagy and the PTEN Signaling Pathway
Source: PLoS Genet. 2013 Oct 3;9(10):e1003845. doi: 10.1371/journal.pgen.1003845 (PMC3789823; doi:10.1371/journal.pgen.1003845)
Supplement: Text S1 — Supporting materials and methods. (DOC) [file pgen.1003845.s004.doc]

**Text S1. Supporting Materials and Methods**

## Supporting Materials and Methods

Animal. *Dat^Cre/+^* mice, *Atg7^flox/flox^* mice, and *Pten^flox/flox^* mice used in this study were generated previously . Genomic DNA extracted from mouse tails were amplified by PCR for genotyping. We used *Atg7* cWT (*Dat^Cre/+^Atg7^+/+^* or *Dat^Cre/+^Atg7^flox/+^*) mice for *Atg7* cKO (*Dat^Cre/+^Atg7^flox/flox^*) mice, and control cWT (*Dat^Cre/+^Pten^flox/+^Atg7^flox/+^*) mice for *Atg7* cKO (*Dat^Cre/+^Pten^flox/+^Atg7^flox/flox^*), *Pten* cKO (*Dat^Cre/+^Pten^flox/flox^Atg7^flox/+^*), *Atg7/Pten* double cKO (*Dat^Cre/+^Pten^flox/flox^Atg7^flox/flox^*) mice (Figure 1A, 7A), as *Dat* heterozygous KO (*Dat^Cre/+^*) mice show some defects in their behavior and physiology .

Mouse genotyping. For PCR genotyping, the following primers were used: 5’-AGATGTTCGCGATTATC-3’, 5’-AGCTACACCAGAGACGG-3’ for *Cre*; 5’-TGCTCTGTGAACTGCCCTGTTT-3’, 5’-TGTTCCTGTGCACTGCCTCATT-3’ for wild-type *Atg7*; 5’-CTTGGGTGGAGAGGCTATTC-3’, 5’-AGGTGAGATGACAGGAGATC-3’ for floxed *Atg7*; 5’-ACTCAAGGCAGGGATGAGC-3’, 5’-AGGGAGGATGAATCTGTGCA-3’ for *Pten*.

Histology. Deeply anesthetized mice were perfused and fixed in 4% paraformaldehyde (PFA) and post-fixed in PFA at 4°C for overnight. Fifty-µm coronal brain sections were made by a vibratome. The antibodies used here were: TH (P60101, Pel-Freez), CRE (PRB106C, Covance), LC3B (NB6001384, Novus), VMAT2 (AB1767, Millipore), Ubiquitin (U5379, Sigma), p62 (03GP62C, ARP), phospho-Akt [S473] (3787, CST), phospho-mTOR [S2448] (2976, CST), and phospho-S6 [S235/236] (#4857, CST). For secondary detection, Cy3- or FITC-conjugated antibodies (Jackson ImmunoRes), Alexa Fluor Dye-conjugated antibodies (Invitrogen), or ABC Elite Kit (Vector) were used.

Measurement of axonal shaft density. Fifty-µm coronal striatal sections were stained by anti-TH antibody (P60101, Pel-Freez) and taken pictures at 63× magnification by a confocal microscope (Carl Zeiss, LSM510). The striatal pictures were binarized and the density of axonal shaft was measured by ImageJ (NIH).

Electron microscopy. Electron microscopic analysis was according to the manufacture’s protocol for general application (Electron Microscopy Sciences, Hatfield, PA). Anesthetized mice were perfused and fixed in PBS containing 4% paraformaldehyde and 0.5% gultaralaldehyde. The brains were post-fixed at 4°C for 2 hours, and 80 μm vibratome sections were made. To enhance penetration, the sections were incubated in 2.5% glycerol and 25% sucrose in PBS for 15 min, mounted in OCT compound, dipped in liquid nitrogen, and immersed in PBS at room temperature . After blocking, they were incubated at 4°C for overnight in buffer containing rabbit anti-TH antibody (1:250, P40101, Pel-Freez). After washing, the sections were incubated in the ultra-small gold-conjugated goat anti-rabbit IgG antibody (#25100, Electron Microscopy Sciences). The sections were post-fixed in 2% gultaralaldehyde/PBS. After washing in distilled water, the silver enhancement was done by using Aurion R-Gent SE-EM (#25521, Electron Microscopy Sciences) for electron microscopy.

AAV2-Cre/GFP infection. Two-µl of 10× diluted AAV2-Cre/GFP viral solution was injected stereotaxically into the right midbrains of anesthetized 2-month-old *Atg7^flox/+^* or *Atg7^flox/flox^* mice. The injection site is 3.1 mm (X-axis), 1.1 mm (Y-axis) and 4.4 mm (Z-axis) from the bregma. For control experiment, AAV2-GFP viral solution was injected into the contralateral site of the midbrain. The mice were perfused in 4% PFA 4- or 8-weeks after the injection, and subjected to immunohistochemical analysis.

Cell size determination. After the TH staining of the midbrain sections by 3,3'-diaminobenzine (DAB), pictures were taken at 40× magnification. The sizes of TH-positive neurons were measured manually by Image-J (NIH). More than 200 TH-positive neurons from 4 mice were analyzed for each group.

Synaptosome preparation. Preparation of the striatal synaptosomal fractions was according to the previous paper . Striatal tissues were taken out from mouse brains, and homogenized in ice-cold Krebs-Ringer Buffer (KRB; 125 mM NaCl, 1.2 mM KCl, 1.2 mM MgSO_4_, 1.2 mM CaCl_2_, 22 mM NaHCO_3_, 1 mM NaHPO_4_, 10 mM Glucose) containing 0.32 M Sucrose. The lysates were centrifuged at 3,000 rpm for 10 min at 4°C. The supernatants were decanted into 1.5 ml tube, and centrifuged at 14,000 rpm for 30 min at 4°C. The pellets were re-suspended in RIPA buffer (#89900, Thermo Scientific) containing protease inhibitor cocktail (P8340, Sigma) and phosphatase inhibitor cocktail (#78420, Thermo Scientific), and subjected to SDS-PAGE and Western Blotting. The antibodies used here were: Actin (ab3280, Abcam), LC3B (#2775, CST), GABARAPL1 (11010-1-AP, Proteintech), EEA1 (ab2900, Abcam), Rab5 (ab18211, Abcam), Rab7 (R4779, Sigma-Aldrich), Cathepsin B (06-480, Upstate), Synapsin I (AB1543, Millipore), Synaptophysin (MAB5258, Millipore), Synaptotagmin I (MAB5200, Millipore), SV2A (ab32942, Abcam), α-Synuclein (sc-7011R, SantaCruz), SNAP25 (ab41455, Abcam), Syntaxin 1A (ab41453, Abcam), Synaptobrevin II (ab3347, Abcam), GAP43 (G9264, Sigma), PSD95 (#75-028, NeuroMab), Gephrin (147-021, Synaptic Systems), AKT (#9271, #9272, #9275, CST), mTOR (#2971, #2974, #2983, CST), phospho-S6K (#9234, CST), phospho-S6 (#2211, CST), 4EBP1 (#9451, #9455, #9644, CST), 4EBP2 (#2845, CST), and phospho-eIF4E (#9741, CST).

Preparation of primary midbrain neurons. Mouse primary midbrain neuron cultures were prepared from E15 embryonic whole midbrains as previously with minor modification . Dissected and trypsinized midbrain cells were plated on poly-D-lysine/laminin-coated 24-well plates at the density of 5.0× 10^4^ cells/well, and cultured in neurobasal medium containing B27 supplement, 200 mM glutamine, 1% fetal bovine serum, 200 µM ascorbic acid, 70 µM uridine, 25 µM 5-fluorodeoxyuridine. Cells were fixed in 4% PFA for 10 min at 5th DIV and stained by anti-TH antibody (P60101, Pel-Freez).

TH-neuron counting. For counting of TH-positive DA neurons, all vibratome sections containing the whole substantia nigra were stained with sheep anti-TH antibody. TH-positive neurons were visualized by DAB. Pictures were taken at 40× magnification. The number of TH-positive DA neurons was counted under the microscopic examination.

HPLC. The determination of dopamine concentration was according to the previous paper . Mice were put to sleep in CO_2_, and the striatal tissues were carefully dissected (9-12 mg). The tissues were put into 500 µl of ice-cold 0.1M percholoric acid solution, homogenized and sonicated on ice. Fifty-µl of homogenates was saved for protein concentration. The homogenates were centrifuged at 14,000 rpm at 4°C for 20 min, and the supernatants were collected. Ortho-phosphoric acid and metabisulfate were added to the supernatants (final concentration, 8.8% and 0.22 mg/ml, respectively). Concentration of dopamine, DOPAC, and HVA in the supernatants was measured by HPLC. The concentrations were standardized by protein concentration.

Mouse behavior. Three-month-old male mice were used for locomotor activity, and the tests were carried out during the dark cycle of their circadian rhythm. The activities of control cWT, *Pten* cKO, *Atg7* cKO, and *Atg7/Pten* double cKO mice (n = 10 ~ 12) were monitored automatically by beam interruption for 30 min in the novel box of 11.0” x 11.0” (MED-OFA-MS, Med Associates). Data was analyzed using Activity Monitor (Med Associates Inc.).

## References

**1. Zhuang X, Masson J, Gingrich JA, Rayport S, Hen R (2005) Targeted gene expression in dopamine and serotonin neurons of the mouse brain. J Neurosci Methods 143: 27-32.**

**2. Komatsu M, Waguri S, Ueno T, Iwata J, Murata S, et al. (2005) Impairment of starvation-induced and constitutive autophagy in Atg7-deficient mice. J Cell Biol 169: 425-434.**

**3. Groszer M, Erickson R, Scripture-Adams DD, Lesche R, Trumpp A, et al. (2001) Negative regulation of neural stem/progenitor cell proliferation by the Pten tumor suppressor gene in vivo. Science 294: 2186-2189.**

**4. Spielewoy C, Biala G, Roubert C, Hamon M, Betancur C, et al. (2001) Hypolocomotor effects of acute and daily d-amphetamine in mice lacking the dopamine transporter. Psychopharmacology (Berl) 159: 2-9.**

**5. Mengual E, Pickel VM (2004) Regional and subcellular compartmentation of the dopamine transporter and tyrosine hydroxylase in the rat ventral pallidum. J Comp Neurol 468: 395-409.**

**6. Teng L, Crooks PA, Sonsalla PK, Dwoskin LP (1997) Lobeline and nicotine evoke [3H]overflow from rat striatal slices preloaded with [3H]dopamine: differential inhibition of synaptosomal and vesicular [3H]dopamine uptake. J Pharmacol Exp Ther 280: 1432-1444.**

**7. MacLeod D, Dowman J, Hammond R, Leete T, Inoue K, et al. (2006) The familial Parkinsonism gene LRRK2 regulates neurite process morphology. Neuron 52: 587-593.**

**8. Xia Z, Dudek H, Miranti CK, Greenberg ME (1996) Calcium influx via the NMDA receptor induces immediate early gene transcription by a MAP kinase/ERK-dependent mechanism. J Neurosci 16: 5425-5436.**

**9. Yang L, Calingasan NY, Chen J, Ley JJ, Becker DA, et al. (2005) A novel azulenyl nitrone antioxidant protects against MPTP and 3-nitropropionic acid neurotoxicities. Exp Neurol 191: 86-93.**
